# Wheat root length and not branching is altered in the presence of neighbours, including blackgrass

**DOI:** 10.1371/journal.pone.0178176

**Published:** 2017-05-24

**Authors:** Jessica A. Finch, Gaëtan Guillaume, Stephanie A. French, Renato D. D. R. Colaço, Julia M. Davies, Stéphanie M. Swarbreck

**Affiliations:** Department of Plant Sciences, University of Cambridge, Cambridge, United Kingdom; Estacion Experimental del Zaidin, SPAIN

## Abstract

The effect of neighbouring plants on crop root system architecture may directly interfere with water and nutrient acquisition, yet this important and interesting aspect of competition remains poorly understood. Here, the effect of the weed blackgrass (*Alopecurus myosuroides* Huds.) on wheat (*Triticum aestivum* L.) roots was tested, since a low density of this species (25 plants m^-2^) can lead to a 10% decrease in wheat yield and herbicide resistance is problematic. We used a simplified growth system based on gelled medium, to grow wheat alongside a neighbour, either another wheat plant, a blackgrass or *Brachypodium dystachion* individual (a model grass). A detailed analysis of wheat seminal root system architecture showed that the presence of a neighbour principally affected the root length, rather than number or diameter under a high nutrient regime. In particular, the length of first order lateral roots decreased significantly in the presence of blackgrass and *Brachypodium*. However, this effect was not noted when wheat plants were grown in low nutrient conditions. This suggests that wheat may be less sensitive to the presence of blackgrass when grown in low nutrient conditions. In addition, nutrient availability to the neighbour did not modulate the neighbour effect on wheat root architecture.

## Introduction

Plants seldom grow in isolation and diverse ecosystems can have greater productivity than monocultures especially when abiotic factors are not limiting primary productivity [1,2]. Within a community, plants interact with each other as demonstrated by shade avoidance syndrome [3], hyponasty in response to touch [4], or exchange of volatile compounds [5]. While the outcome of these plant to plant interactions can be beneficial (*e*.*g*., facilitation due to nutrient release) or neutral, in agro-ecosystems detrimental effects (*e*.*g*., competition, parasitism) [6,7] can cause severe yield loss.

*Alopecurus myosuroides* Huds. (blackgrass) is a major weed for winter cereals in Europe, that can cause substantial yield loss for wheat [8,9] and oilseed rape (*Brassica napus*)[10]. Blackgrass has become more abundant in agro-ecosystems since 1867 [11] and more potent against wheat as high nutrient conditions have been favoured [12]. The development of herbicide resistance [9] and a European Union directive to prioritize non-chemical methods of pest management [13] necessitate the testing of different agronomic practices, but their efficiencies in controlling blackgrass have remained highly variable [14]. In the field, 25 blackgrass individuals per m^2^ can lower wheat yield by 10% [8] thus suggesting that weed plants have a strong effect on crop growth (typically wheat plants are grown at a density of 350–500 seeds m^-2^) and yet the mechanism underlying this strong effect is not understood. In addition, wheat varieties have shown variable competitive levels against blackgrass ([14]; and references therein). Investigating the mechanism of the blackgrass effect or varietal differences in field is also problematic because of environmental variations and thus far no *in vitro* method has been developed.

In the soil, roots compete with roots from neighbouring plants (either of their own or different species and genotypes) for anchorage, nutrients and water. The root to root interactions already described are species-specific [15] and can lead to growth increase [16,17], or decrease [17–21] or change in growth direction [22–25]. While some aspects of root interactions relate directly to the availability of nutrients in the vicinity of a competitor, recent evidence suggests that roots may be able to detect neighbouring roots independently of the nutrient level [24]. Indeed, root exudates have been implicated as potential signals detected by neighbouring roots [23,26,27]. While allelopathic compounds that can induce cell death have been characterized [28–30], no specific compounds or group of compounds have thus far been directly implicated in the more subtle inter-species root interactions that can modulate root system architecture (RSA). Based on our current understanding of the mechanism underlying root to root interactions, it is likely that for many species this phenomenon can also be observed using an artificial growth system that allows diffusion of the signalling compounds, as it was the case for rice (*Oryza sativa*) [24].

Given that root growth and maintenance is costly for plants, the ability to respond to the presence of roots from competing neighbours should give a strong advantage and be widespread among plant species, including cereal crops. Evidence for this process is seen in rice, where the roots from individuals of the same genotype overlap more than those from different genotypes [24]. Further analyses indicated that this mechanism is different from the detection of an object by physical contact, and implicates the detection of root exudates. Furthermore, maize (*Zea mays*) total root length shows genotypic differences in response to competition from soybean (*Glycine max*) [31]. However, very little is known about the effect of neighbours on root architecture of wheat (*Triticum aestivum* L.), which is a major crop worldwide, and for which production reached above 729 Mt in 2014 (http://faostat.fao.org). In addition, thus far no experimental system has been adapted specifically to investigate the neighbour effect on wheat root system architecture.

The aim of this study was to establish an experimental system that would enable detailed analysis of wheat seminal root system architecture (RSA) in the presence of neighbours (including blackgrass), an aspect of the weed effect that has seldom been investigated [32]. In order to test this new experimental system we selected a wheat cultivar (Hereward) that is known by growers as being particularly susceptible to blackgrass [14]. Hereward plants were grown in the presence of neighbours that were either another Hereward plant, a blackgrass or a *Brachypodium dystachion* L. (*Brachypodium*) individual. *Brachypodium* is a temperate, annual grass and a model for monocotyledenous species [33], having a fully sequenced genome [34,35] that was used here as a non-wheat and non-weed control. Finally, experiments under high and low nutrient levels were conducted that tested the hypothesis that nutrient availability to the neighbours would modulate neighbor effects on wheat.

Using this new experimental system, specific wheat root responses to the presence of neighbour were identified for the first time. In particular, we found that the presence of a neighbour could significantly alter the length but not the number or diameter of seminal and lateral roots of wheat plants grown under high nutrient conditions. While the presence of wheat neighbours could shorten seminal roots, the presence of blackgrass or *Brachypodium* had a stronger effect on laterals. In addition, the wheat response to a neighbour measured in high nutrient conditions was not necessarily present in low nutrient conditions while the nutrient availability to the neighbour had no significant effect.

## Results

### In high nutrients, wheat seminal roots are shorter in the presence of a wheat neighbour

**Fig 1:**
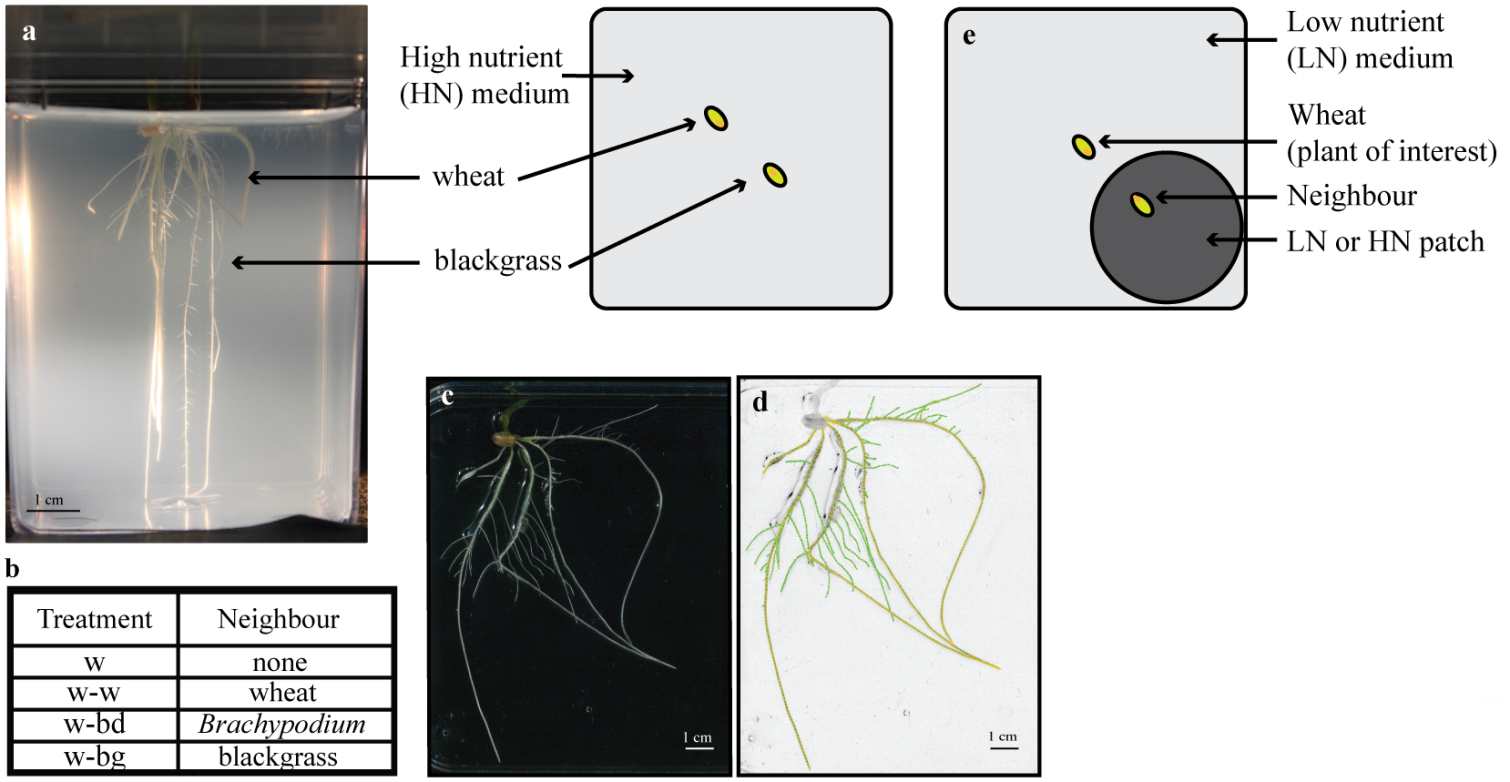
Experimental setup for investigating the effect of a neighbour on wheat RSA. (a) Wheat plant grown in homogeneous MS medium with 0.12% (w/v) phytagel alongside a blackgrass individual. (b) Focal wheat plants were grown under different treatments. (c) Wheat roots system extracted from the medium, laid flat on a transparent plate for scanning. (d) Inverted images with dark roots on a white background, and traced using SmartRoot [36]. (e) Second experimental setup, with the focal (*i*.*e*., plant of interest) wheat grown in low nutrient (LN) medium and its neighbour (either w, wheat or bd, *Brachypodium* or bg, blackgrass) grown in a patch of LN or high nutrient (HN) medium.

To investigate the effect of a neighbour on wheat RSA, wheat plants were grown alone (w) or in the presence of another individual of wheat (w-w), blackgrass (w-bg) or *Brachypodium* (w-bd) for 11–13 days in high nutrient (HN) medium (Fig 1a and 1b, *n* = 11–12 plants in three independent repeats). The focal wheat seedling (w) was added simultaneously with the wheat neighbour or when the blackgrass or *Brachypodium* neighbour was already 11 days-old, to achieve a similar final biomass for a wheat or blackgrass neighbour. At the end of the experiment, roots were extracted from the medium for a detailed analysis of RSA parameters (Fig 1c and 1d, Table 1).

**Table 1:** Description of root system architecture parameters.

Root trait	Description
**Seminal root length (cm)**	Primary root (extending from the radicle) could not be distinguished from the embryonic crown roots (emerging from embryonic nodes), thus all are called seminal roots here.
**Length of the ramified region (cm)**	Section of the root (between the first and last lateral) from which lateral root emergence was detected.
**Lateral root density in the ramified region (number of roots cm**^**-1**^**)**	Number of lateral roots in the ramified region. Lateral root primordia that were apparent (but for which emergent length could not be measured) were also counted.
**Lateral root length (cm)**	Length measurement for first or second order laterals.
**Cumulative length (cm)**	Sum of length from all roots in one category either seminals or laterals.
**Relative lateral root position**	The distance from the base of the root to the point of emergence of the lateral divided by the length of the root.
**Root diameter (cm)**	Diameter of the root averaged along the root length.

**Fig 2:**
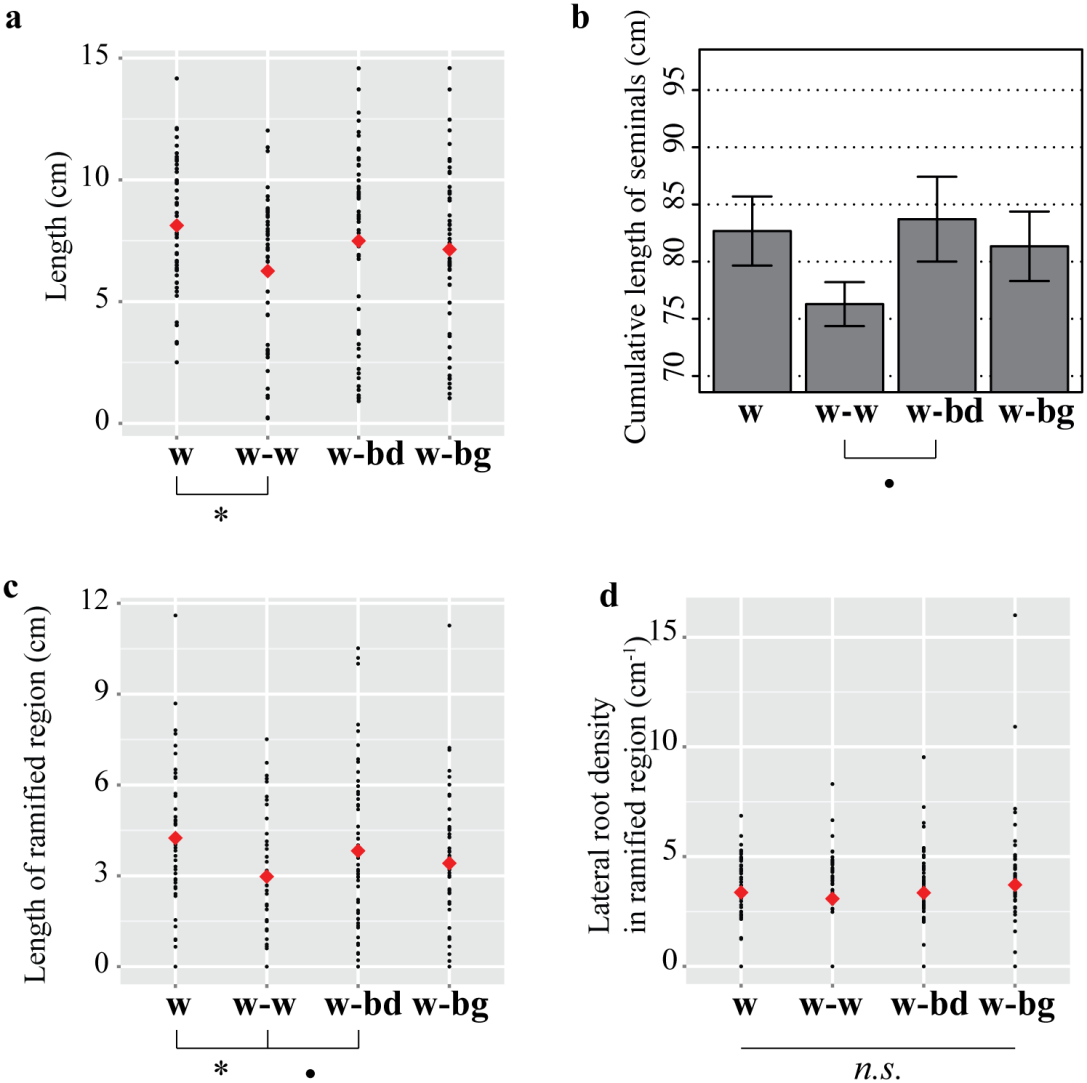
Seminal roots characteristics of wheat grown in HN conditions and in the presence of a different neighbour (w, wheat alone; w-w, wheat against wheat; w-bd, wheat against *Brachypodium*; w-bg, wheat against blackgrass). (a) Seminal length for individual roots (black filled circles) and treatment mean (red diamonds). (b) Cumulative length of seminals shown as mean ± se. (c) Length of the ramified region. (d) Lateral root density in the ramified region. Data obtained from 11–12 plants per treatment in three separate experiments;^**.**^ *p* < 0.1, * *p* < 0.05, *n*.*s*., no significant.

We first considered how a neighbour’s presence affects wheat seminal roots (number, length, diameter) and their density of laterals in HN conditions. The mean number of seminal roots remained constant amongst wheat plants grown on their own (w: mean ± se, seminal count, 4.64 ± 0.24), alongside another wheat plant (w-w: seminal count, 5.00 ± 0.40), a blackgrass (w-bg: seminal count, 5.09 ± 0.21) or a *Brachypodium* individual (w-bd: seminal count, 5.17 ± 0.44; Student’s *t*-tests, *n*.*s*.). To account for multiple measurements per individual plant linear mixed effect model (lme) analyses were conducted. These showed that the presence of another wheat plant (w-w) led to a significant (lme, *p* < 0.05) 23% decrease in the length of the focal wheat seminal roots (mean length ± se 6.3 ± 0.4 cm) compared to wheat grown alone (8.1 ± 0.4 cm), while no significant difference was found in root length between wheat plants grown alone (w) and wheat grown with blackgrass (w-bg) or *Brachypodium* (w-bd), though the difference between w-w and w-bd was significant at the 10% level (*p* = 0.088, Fig 2a). The overall effect of a neighbour on the length of wheat seminal roots was not significant (Fig 2a, ANOVA lme, F_(3,41)_ = 1.65, *p* = 0.19), perhaps because of the effect of blackgrass and *Brachypodium* was similar and not significant. Cumulative length of seminal roots (Fig 2b) was not significantly different when wheat plants were grown alone or in the presence of a neighbour, either wheat (Student’s *t*-test, *p* = 0.15), blackgrass (*p* = 0.76) or *Brachypodium* (*p* = 0.81), perhaps because the changes in mean seminal length are too subtle to affect the overall cumulative length. The seminal root diameter of the focal wheat plants was not significantly affected by the presence of neighbour (S1a Fig, ANOVA lme, F_(3,41)_ = 0.31, *p* = 0.81).

As for seminal root length, the length of the ramified region (Fig 2c) was shorter in wheat with a wheat neighbour (3.0 ± 0.3 cm) compared to wheat grown alone (4.2 ± 0.3 cm, lme, *p* < 0.05), but not with blackgrass (3.4 ± 0.3 cm) or *Brachypodium* (3.8 ± 0.4 cm). The overall effect of the different treatments was not significant (ANOVA lme, F_(3,41)_ = 1.77, *p* = 0.17), again perhaps because of the similar effect of blackgrass and *Brachypodium*. There was no significant effect of neighbour identity on the relative location of the first lateral root (closest to the base of the seminal, F_(3,41)_ = 0.65, *p* = 0.58, S2a Fig) or the last lateral root (furthest to the base of the seminal, F_(3,41)_ = 0.99, *p* = 0.41, S2b Fig). The density of lateral roots in the ramified region was not significantly affected by a neighbour (Fig 2d, F_(3,41)_ = 0.55, *p* = 0.64). Overall, our data show that only a neighbouring wheat’s presence affected wheat seminal roots, with only length affected.

### In high nutrients, wheat lateral roots were shorter in the presence of a neighbour

**Fig 3:**
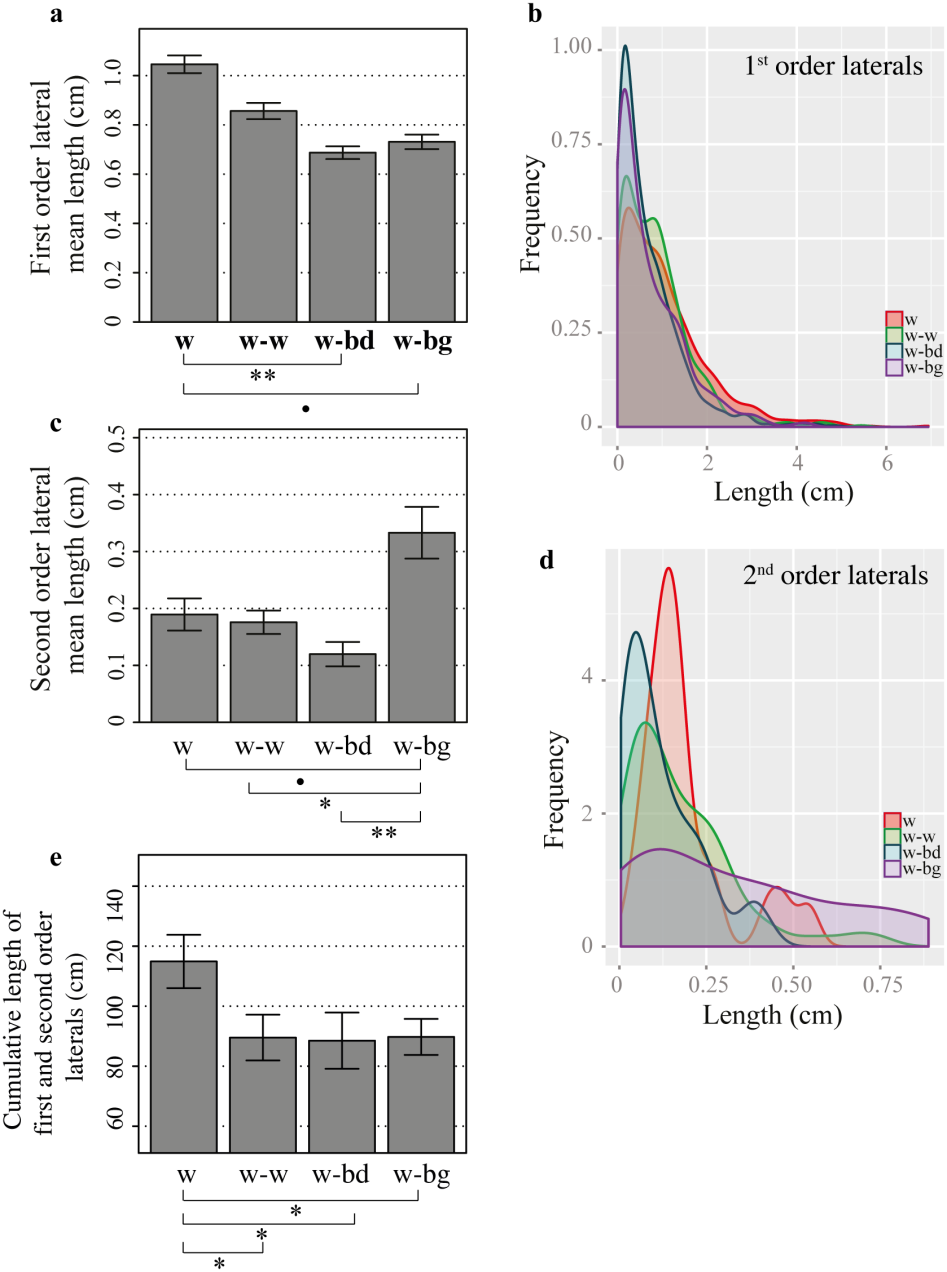
Characteristics of lateral roots of wheat grown in high nutrient conditions and in the presence of different neighbours (w, wheat alone; w-w, wheat against wheat; w-bd, wheat against *Brachypodium*; w-bg, wheat against blackgrass). (a) Mean length for first order laterals ± se. (b) Frequency distribution of first order lateral root length. (c) Mean length for second order laterals ± se. (d) Frequency distribution of second order lateral root length. (e) Mean cumulative length of first and second order laterals ± se. Data obtained from 11–12 plants per treatment in three separate experiments;^**.**^ *p* < 0.1, * *p* < 0.05, ** *p* < 0.01.

We then considered how a neighbour’s presence affects wheat first order lateral roots (*i*.*e*. those emerging from the seminal) and second order laterals (*i*.*e*. those emerging from first order laterals). First order laterals of focal wheat plants were on average shorter in the presence of a neighbour (Fig 3a, ANOVA lme, F_(3,41)_ = 2.97, *p* < 0.05). This effect on lateral length was more pronounced in the presence of *Brachypodium* (mean length ± se, 0.69 ± 0.03 cm, a decrease of 34.3% compared to wheat alone, lme, *p* < 0.05) or blackgrass (mean length ± se, 0.73 ± 0.03 cm, a decrease of 30.5% compared to wheat alone, *p* = 0.059). A wheat neighbour had a weaker effect on lateral mean length than *Brachypodium* or blackgrass (mean length 0.86 ± 0.03 cm, a decrease of 18.1% compared to wheat alone, *p* = 0.23). This lesser effect of a wheat neighbour could perhaps be linked to the fact that in the w-w treatment, roots from the focal wheat grow alongside its neighbour, whereas in w-bd and w-bg treatments, wheat roots grow in an already colonised rhizosphere.

Frequency distribution of lateral root length showed that a higher proportion of lateral roots were shorter than 0.5 cm when wheat was grown in the presence of blackgrass or *Brachypodium* than in the presence of wheat or alone (Fig 3b). There was no difference in the effect of blackgrass and *Brachypodium* on the frequency distribution of lateral root length (Kolmogorov-Smirnov test, *n*.*s*.) but both had a significant effect compared to wheat or the absence of a neighbour (Kolmogorov-Smirnov test, *p* < 0.05). Similar to seminals, root diameters of first order laterals remained unaffected by the presence of a neighbour (S1b Fig, ANOVA lme, F_(3,41)_ = 0.31, *p* = 0.79).

Few second order laterals emerged (total number from three separate experiments, w, *n* = 22; w-w, *n* = 58; w-bg, *n* = 38; w-bd, *n* = 26) and there was no significant difference in their density amongst the different treatments (data not shown, ANOVA lme, F_(3,41)_ = 0.41, *p* = 0.75), nor in their diameter (S1c Fig, F_(3,22)_ = 0.24, *p* = 0.86). Although the overall effect of neighbour was only significant at the 10% level (ANOVA lme, F_(3,22)_ = 2.92, *p* = 0.056), mean length of second order laterals was higher in the presence of blackgrass compared to other neighbours (Fig 3c). The distribution of second order lateral root lengths showed that a lower proportion of roots under 0.25 cm was present in wheat plants grown in the presence of blackgrass compared to wheat plants grown alone, in the presence of another wheat plant or a *Brachypodium* (Fig 3d, Kolmogorov-Smirnov test, *p* < 0.05).

The cumulative length of lateral roots (both first and second order, Fig 3e) was decreased by 22% in the presence of another wheat plant (Student’s *t*-test, *p* < 0.05), 23% in the presence of *Brachypodium* (*p* < 0.05) and 22% in the presence of blackgrass (*p* < 0.05) compared to wheat alone. There was no significant difference amongst plants grown with a neighbour (wheat vs. blackgrass, *p* = 0.98; blackgrass vs. *Brachypodium*, *p* = 0.73). Overall, a neighbour’s presence significantly affected lateral roots but in contrast to the seminal roots, the effect on laterals was due to a non-wheat neighbour (*i*.*e*., blackgrass or *Brachypodium*).

### Neighbours affect wheat seedling RSA at constant biomass in high nutrient conditions

**Fig 4:**
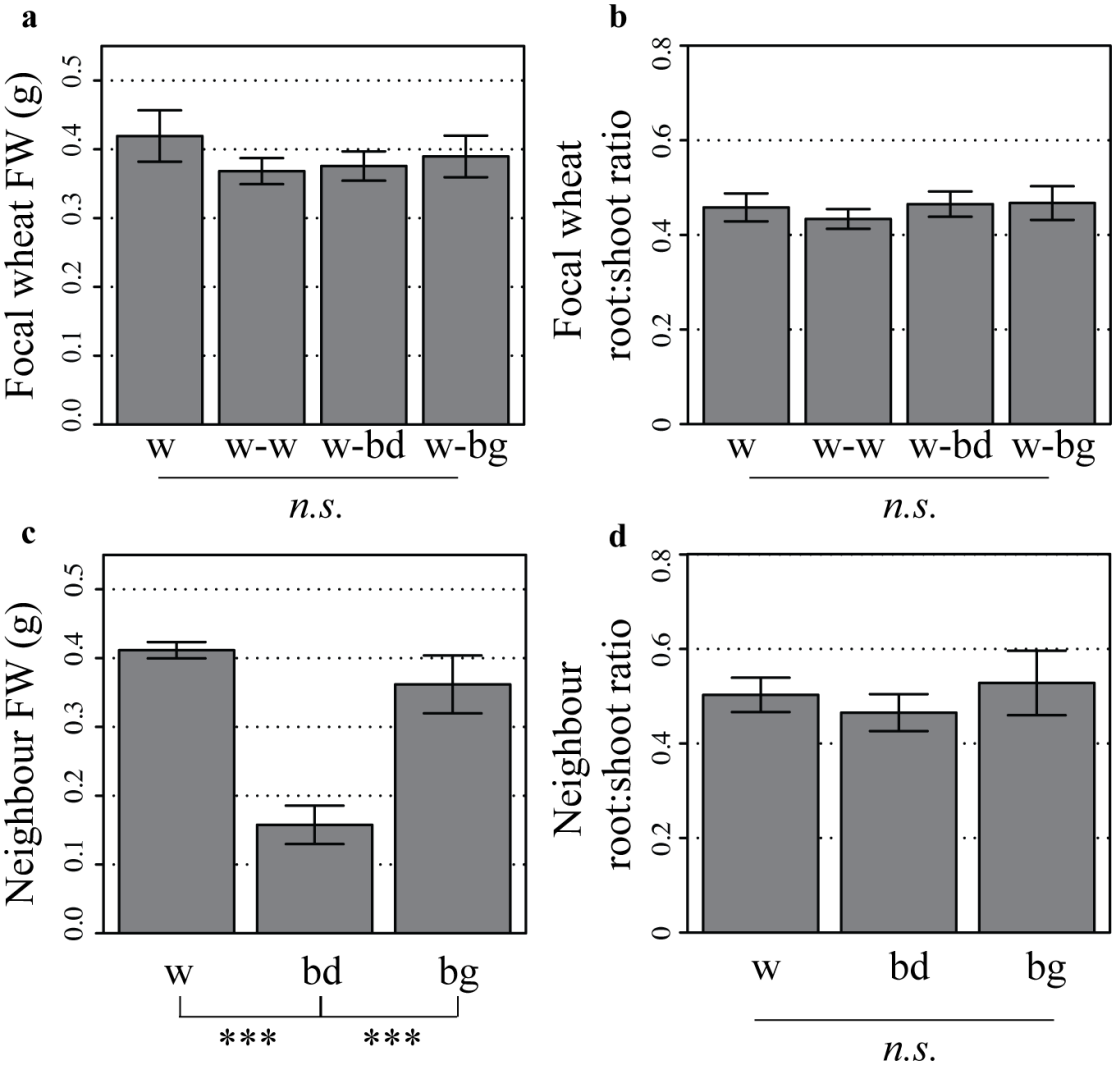
Total fresh weight of focal wheat plants and neighbours grown for 11–13 days in high nutrient conditions. (a) Total fresh weight (roots and shoots) and (b) root to shoot ratio of the focal wheat plant in the presence of different neighbours (w, wheat alone; w-w, wheat against wheat; w-bd, wheat against *Brachypodium*; w-bg, wheat against blackgrass). (c) Total fresh weight and (d) root to shoot ratio of neighbour plants (w, wheat; bd, *Brachypodium*; bg, blackgrass). Data shown as mean ± se, and obtained from the same plants as in Figs 2 and 3 (*n* = 11–12, in three separate experiments);^**.**^ *p* < 0.1, * *p* < 0.05, ** *p* < 0.01 and *** *p* < 0.001, *n*.*s*., not significant.

As the presence of a neighbour has been shown to affect biomass accumulation [6], biomass of the wheat focal plants was measured at the end of the experiments. Wheat fresh weight (FW, Fig 4a) was not significantly affected by the presence of another wheat plant (Student’s *t*-test, *p* = 0.21), a *Brachypodium* individual (*p* = 0.28) or a blackgrass individual (*p* = 0.46). Similarly, the root to shoot ratio (Fig 4b) of the focal wheat plant was maintained in the presence of another wheat plant (Student’s *t*-test, *p* = 0.55), a *Brachypodium* (*p* = 0.9) or a blackgrass individual (*p* = 0.76). Therefore, with the focal wheat biomass constant, neighbours could induce significant changes in wheat RSA, that would have been masked if plant weight alone had been considered.

Biomass of neighbours was also measured at the end of experiments. That of *Brachypodium* was overall much lower than that of the wheat neighbours or blackgrass (Student’s *t*-test, *p* < 0.001, Fig 4c), while there was no difference between the wheat and blackgrass neighbour (*p* = 0.26). Root to shoot ratio (Fig 4d) was similar between wheat and blackgrass (*p* = 0.73) or wheat and *Brachypodium* (*p* = 0.61). It is interesting to note that while *Brachypodium* was smaller at the end of the experiment than blackgrass or a wheat neighbour, it could affect wheat RSA to a similar level, over this timecourse.

### In low nutrients, wheat seminal roots remain unaffected by the presence of a wheat neighbour

Next, we examined whether a wheat plant in low nutrient (LN) conditions showed a similar response to that seen in high nutrient (HN) (Figs 2 and 3) and tested whether nutrient availability to the neighbour affected the outcome. An individual wheat plant was grown in LN medium while the neighbouring plant (wheat, blackgrass or *Brachypodium*) was placed in a patch of LN or HN medium (Fig 1e).

**Fig 5:**
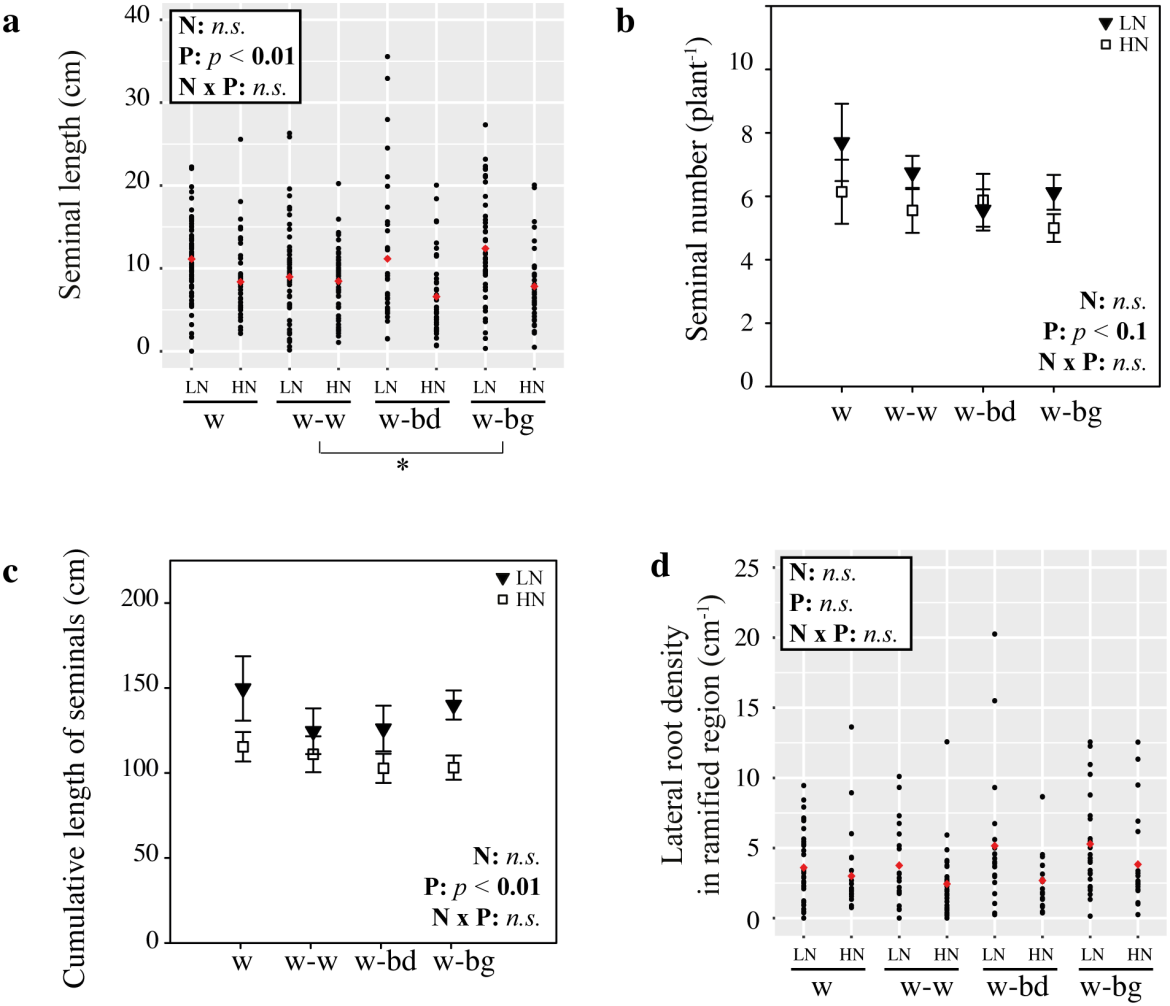
Seminal root characteristics of wheat grown in LN conditions, in the presence of a different neighbour with a LN or HN patch. The focal wheat plant was grown in low nutrient (LN) medium while its neighbours (w, wheat; bd, *Brachypodium*; bg, blackgrass) were grown in a patch of low (LN, black triangles) or high nutrient (HN, white squares) medium. (a) Seminal root length, individual root data (black filled circles) and treatment means (red diamonds). (b) Seminal root number mean ± se. (c) Cumulative length of seminals, mean ± se. (d) Lateral root density in the ramified region, individual root data (black filled circles) and treatment means (red diamonds). Data shown are from three separate experiments, *n* = 7–10 plants. Significance levels of main factors (N, neighbour, P, patch) and their interactions (N x P) are shown. Significant differences amongst neighbours are indicated below the x-axis,^**.**^ *p* < 0.1, * *p* < 0.05, *n*.*s*., not significant.

In LN, seminal roots were significantly longer compared to the HN treatment (ANOVA lme, F_(1,70)_ = 15.5, *p* < 0.05). While wheat seminal roots in homogeneous HN conditions were shorter with a wheat neighbour (Fig 2a), overall in LN these were unaffected by neighbours (ANOVA lme, F_(3,56)_ = 1.08, *p* = 0.36). However, there is no significant nutrient x neighbour interaction (ANOVA lme, F_(3,70)_ = 0.93, *p* = 0.4), perhaps because the effect seen under HN is only due to the presence of a wheat neighbour. In LN, a wheat neighbour had no effect (Fig 5a, lme, *p* = 0.33) compared to wheat alone, however the effect of blackgrass was significantly different to that of a wheat neighbour (lme, *p* <0.05).

In LN, neighbours had no significant effect on seminal root number (Fig 5b, ANOVA, F_(3,56)_ = 0.81, *p* = 0.49), diameter (S1d Fig, ANOVA lme, F_(3,56)_ = 0.33, *p* = 0.8), cumulative length (Fig 5c, ANOVA, F_(3,56)_ = 1.33, *p* = 0.27) or lateral root density (Fig 5d, F_(3,56)_ = 0.64, *p* = 0.59). Overall in LN, neighbours had no effect on wheat seminal roots, which differs from the neighbour effect on wheat roots grown in HN.

There was also no significant effect of the interaction between patch nutrient level and the presence of a neighbour on seminal length (ANOVA lme, F_(3,56)_ = 1.09, *p* = 0.36), seminal number (ANOVA, F_(3,56)_ = 0.43, *p* = 0.73), diameter (ANOVA lme, F_(3,56)_ = 0.55, *p* = 0.65), cumulative length (F_(3,56)_ = 0.41, *p* = 0.75), or first order lateral root density (F_(3,56)_ = 1.0, *p* = 0.40). This suggests that nutrients available to the neighbour do not significantly modulate the neighbour effect on wheat seminal roots.

### Mean wheat lateral roots remain unaffected by the presence of a non-wheat neighbour in low nutrients

**Fig 6:**
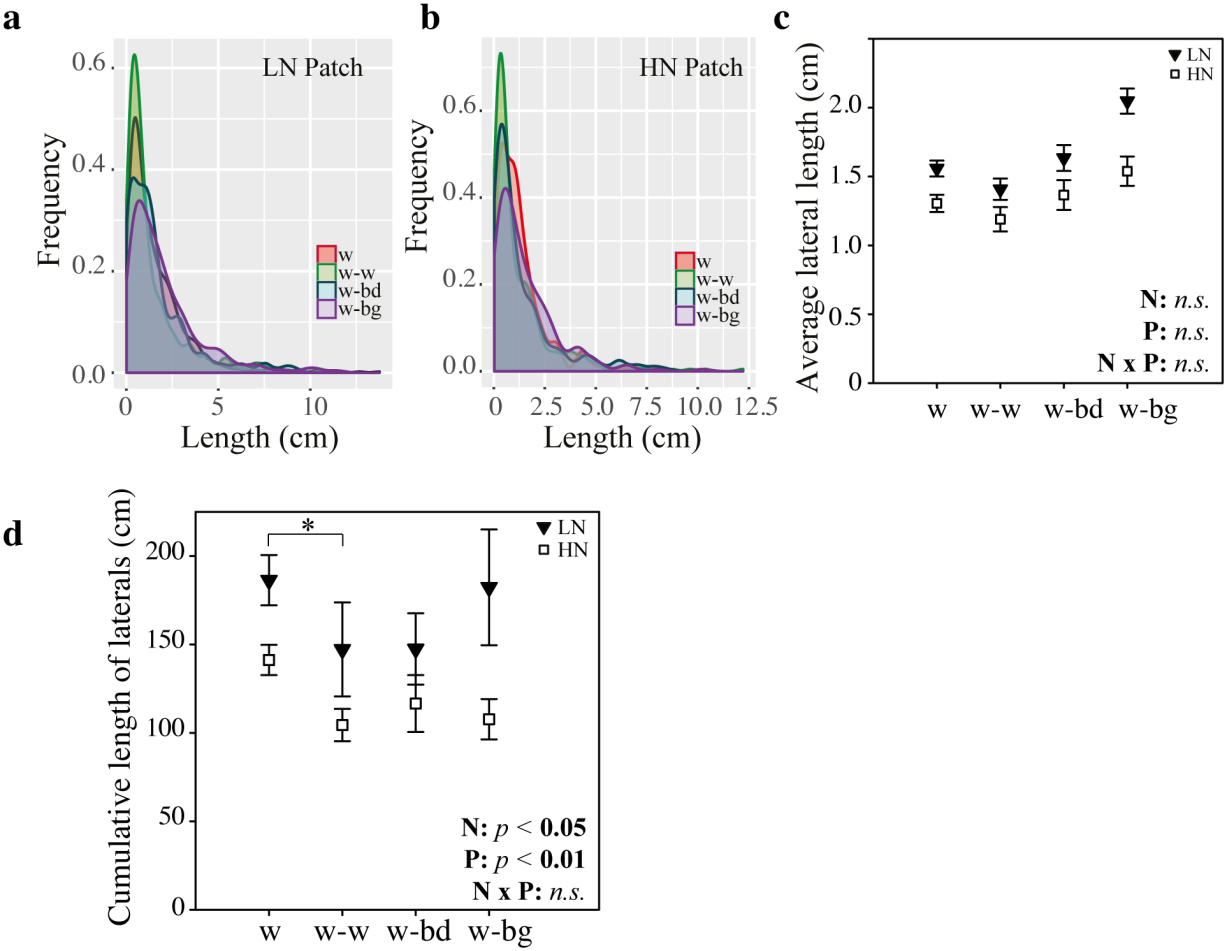
First order lateral roots characteristics of wheat grown in LN conditions, in the presence of a different neighbour with a LN or HN patch. The focal wheat plant was grown in low nutrient (LN) medium while its competitors (w, wheat; bd, *Brachypodium*; bg, blackgrass) were grown in a patch of low (LN, black triangles) or high nutrient (HN, white squares) medium. (a) Frequency distribution of laterals length in LN with LN patch. (b) Frequency distribution of laterals length in LN with HN patch. (c) Length of first order laterals, mean ± se. (d) Cumulative length of laterals. Data shown are from three separate experiments, *n* = 7–10 plants. Significance levels of main factors (N, neighbour, P, patch) and their interactions (N x P) are shown. Significant differences amongst neighbours are indicated below the x-axis,^**.**^ *p* < 0.1, * *p* < 0.05, *n*.*s*., not significant.

In LN, lateral roots were longer than in HN conditions (F_(1,70)_ = 28.9, *p <* 0.001). The suppressive effect of neighbours on lateral roots observed in homogeneous HN conditions (Fig 3a and 3b) did not occur when the wheat focal plant grew in LN conditions with either a LN or HN patch (Fig 6a, 6b and 6c, ANOVA lme, F_(3,54)_ = 1.48, *p* = 0.23). However, there was no statistically significant interaction between nutrient level and the presence of neighbour in terms of average length of roots (F_(3,70)_ = 1.4, *p* = 0.24).

This distribution of lateral root lengths was also different in LN compared to HN (Fig 6a and 6b). With a LN patch, the proportion of laterals shorter than 0.5 cm was not higher in the presence of blackgrass or *Brachypodium* (as in Fig 3b), rather it was lower (Kolmogorov-Smirnov tests, *p* < 0.05). With a HN patch, the treatment showing the highest proportion of shorter laterals was w-w (Kolmogorov-Smirnov test, *p* < 0.001), the effect of *Brachypodium* was similar to that of wheat (Kolmogorov-Smirnov test, *p* = 0.35), whereas the proportion of shorter laterals was lower in the presence of blackgrass (Kolmogorov-Smirnov test, *p* < 0.001). In LN, differences in lateral length distribution did not lead to significant changes in mean lateral root length in the presence of a neighbour (lme, F_(3,54)_ = 1.48, *p* = 0.23).

There was a significant effect of neighbour on cumulative lateral root length with both HN and LN patches (Fig 6d, F_(3,54)_ = 2.82, *p* < 0.05). In particular, the addition of another wheat plant significantly lowered the cumulative lateral root length (Fig 6d, TukeyHSD, *p* < 0.05), both with a LN (21% decrease) and HN patch (26% decrease). With a LN patch, the wheat response to the presence of a blackgrass neighbour was much lower (2.2% decrease), but with a HN patch it was similar to that of wheat (23.7% decrease). Overall there was no significant effect of blackgrass (TukeyHSD, *p* = 0.37) or *Brachypodium* (TukeyHSD, *p* = 0.22) on cumulative lateral root length. This is different from the response measured under homogeneous HN conditions where the presence of any neighbour affected cumulative lateral root length (Fig 2b), however, the interaction of nutrient level and neighbour is not statistically significant (F_(3,70)_ = 0.176, *p* = 0.9). There was no significant effect of neighbour identity on the relative location of the first lateral root (closest to the base of the seminal, F_(3,54)_ = 2.73, *p* = 0.05, S2c Fig) or the last lateral root (furthest to the base of the seminal, F_(3,54)_ = 0.58, *p* = 0.63, S2d Fig). Very few second order laterals could be observed when wheat plants were grown in LN conditions (with a LN or HN patch), which means that no treatment effect (either from the presence of a nutrient patch or neighbour identity) could be assessed for significance. There was also no significant effect of the interaction between patch nutrient level and neighbour on first order laterals mean length (ANOVA lme, F_(3,54)_ = 0.09, *p* = 0.96), diameter (ANOVA lme, F_(3,54)_ = 0.39, *p* = 0.76), or cumulative length (ANOVA, F_(3,54)_ = 0.27, *p* = 0.85).

### High nutrient patch lead to shortening of seminal and lateral wheat roots

Considering the effect of patch nutrient level on its own may give information relating to the role of nutrient availability in mediating the neighbour effect. The number of seminal roots tended to be lower with a HN patch (Fig 5b), but was only significant at the 10% level (ANOVA, F_(3,56)_ = 3.987, *p* = 0.051). Seminal roots were on average shorter in the presence of a HN patch compared to a LN patch (Fig 5a, ANOVA lme, F_(1,56)_ = 10.13, *p* < 0.05). Seminal root diameter (S1d Fig) remain unaffected by the presence of a patch (ANOVA lme, F_(1,56)_ = 0.32, *p* = 0.58). Cumulative length of wheat seminal roots was significantly lower in the presence of a HN patch compared to a LN patch (Fig 5c, ANOVA, F_(1,56)_ = 10.17, *p* < 0.01). Lateral root density (Fig 5d) was also maintained under the nutrient levels in the patch (ANOVA lme, F_(1,56)_ = 0.024, *p* = 0.88).

Lateral roots tended to be shorter in the presence of a HN patch compared to LN patch but this was not significant (Fig 6c, F_(1,54)_ = 1.72, *p* = 0.19). Cumulative length of wheat lateral roots was significantly lower in the presence of a HN patch (Fig 6d, F_(3,54) =_ 9.49, *p* < 0.05). Overall, the addition of a HN patch (which could increase nutrient availability to the focal wheat plant) also lead to the shortening of both seminals and laterals. This is also in agreement with our observations that wheat roots were longer when grown in LN (Figs 5a and 6c) compared to HN (Figs 2a and 3a). Interestingly, the strongest neighbour effect could be seen when plants were grown in HN where roots were shortest. Our data suggest that while the presence of a HN patch affects wheat RSA, it does not modulate the neighbour effect.

### In low nutrient conditions wheat biomass is decreased in the presence of a neighbour

**Fig 7:**
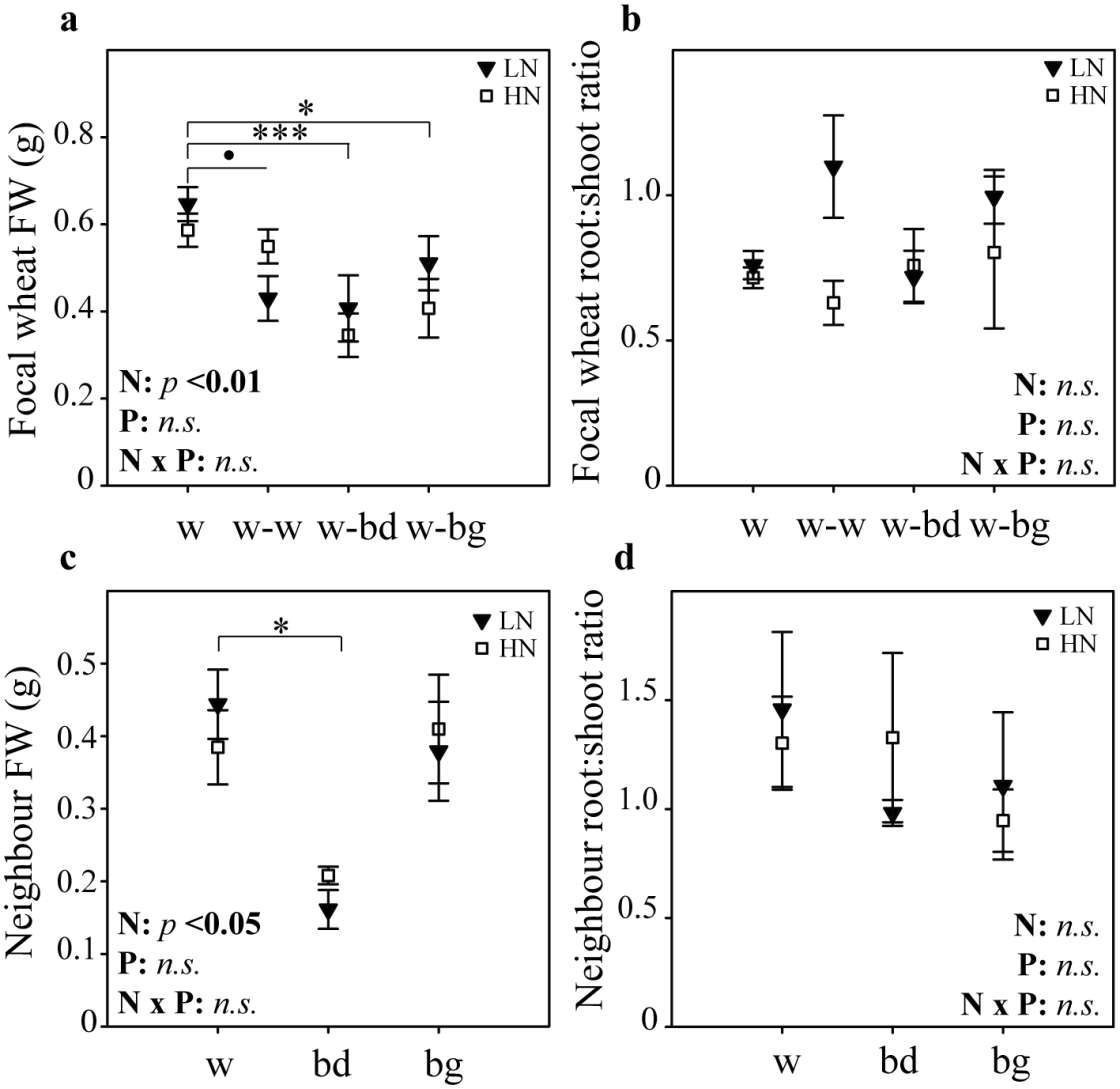
Total fresh weight and root to shoot ratio of wheat and neighbours grown in heterogeneous nutrient conditions. The focal wheat plant was grown in low nutrient (LN) medium while its neighbours (w, wheat; bd, *Brachypodium*; bg, blackgrass) were grown in a patch of low (LN, black triangles) or high nutrient (HN, white squares) medium. (a) FW and (b) root to shoot ratio of the focal wheat plant grown in different competitive conditions (w, wheat alone; w-w, wheat against wheat; w-bd, wheat against *Brachypodium*; w-bg, wheat against blackgrass). (c) FW and (d) root to shoot ratio of neighbours. Data shown as mean ± se, *n* = 7–10 plants from three separate experiments, except for root to shoot ratio of plant of interest where *n* = 5–10 from two separate experiments. Significance levels of main factors (N, neighbour, P, patch) and their interactions (N x P) are shown. Significant differences amongst neighbours are indicated below the x-axis,^**.**^ *p* < 0.1, * *p* < 0.05, ** *p* < 0.01, *** *p* < 0.001, *n*.*s*., not significant.

While neighbours did not significantly affect wheat FW in homogeneous HN conditions (Fig 4a), they had a significant negative effect on wheat FW in LN in the presence of either a LN or HN patch (Fig 7a, ANOVA, F_(3,55)_ = 7.99, *p* < 0.01). In particular, the presence of blackgrass (Tukey HSD, *p* < 0.05) or *Brachypodium* (Tukey HSD, *p* < 0.01), but not wheat (Tukey HSD, *p* = 0.059) induced a reduction in wheat FW compared to wheat alone. Blackgrass and *Brachypodium* had a similar effect on wheat FW (Tukey HSD tests, *n*.*s*.). The FW response mirrors to some extent the pattern of responses seen for cumulative length of seminals (Fig 5c) and laterals (Fig 6d), however more subtle responses, especially in terms of lateral roots, are masked. There was no significant interaction between overall nutrient level and the presence of a neighbour on wheat FW (ANOVA, F_(3, 71)_ = 0.014, *p* = 0.99). There was no significant effect of nutrient patch; the neighbour effect was similar in HN and LN patches (F_(1,55)_ = 0.422, *p* = 0.52).

Root to shoot ratio of wheat plants (Fig 7b) was generally unaffected by neighbours (F_(3,37)_ = 0.728, *p* = 0.54) or patch nutrient level (F_(1,37)_ = 2.63, *p* = 0.11). This suggests that in terms of biomass accumulation a focal wheat plant grown in LN (with either HN or LN patch) is more susceptible to a neighbour, and that overall, both shoot and root were equally suppressed by the neighbour in LN.

Similar to the experiment conducted in homogeneous HN (Fig 4c and 4d), there was a significant difference in total, final biomass of neighbours (Fig 7c, ANOVA, F_(2,39)_ = 4.55, *p* < 0.05) in LN (with either HN or LN patch). As before, the difference amongst neighbours was driven by *Brachypodium* which was significantly smaller than the wheat neighbour (Tukey HSD, *p* < 0.05), and there was no difference between wheat and blackgrass (Tukey HSD, *p* = 0.52). There was overall no significant effect of patch nutrient level (F_(1,39)_ = 0.28, *p* = 0.66). The root to shoot ratio was similar amongst neighbours (F_(2,39)_ = 1.3, *p* = 0.28) and remained unaffected by patch nutrient level (Fig 7d, F_(1,39)_ = 0.19, *p* = 0.67). Similar to the experiment in HN, the wheat RSA response to the presence of *Brachypodium* (*e*.*g*., cumulative lateral length, Fig 6d) was similar to the presence of a wheat or blackgrass neighbour even though *Brachypodium* overall biomass was much lower.

## Discussion

Modern wheat varieties have been bred for many traits including grain quality, short stature, and resistance to pests and disease [37,38]. Root-related traits have been comparatively neglected, partly due to the difficulty in studying them [39,40]. The aim here was to establish an experimental system that would enable detailed analysis of wheat root architecture in the presence of neighbours and to investigate the effect of blackgrass on wheat RSA. A simple non-soil growth system (under sterile conditions) allowed new insights to be gained on the effect of blackgrass on wheat plants.

Here, wheat roots grew inside a gelled medium supplemented with nutrients as opposed to the surface as described previously [41,42], since growth patterns can differ [43]. Our system offers several advantages. (1) Roots can easily be extracted without damaging the fine laterals. (2) The size of the growth boxes allowed roots to intermingle, as they would in soil, thus encouraging interactions amongst neighbours. (3) Root exudates as likely mediators of neighbour-induced changes in RSA can diffuse between plants. Our system also holds some limitations as our observations only cover the early stages of seedling development and may be too simplistic a model of the rhizosphere. Further analyses are required to firmly link the pattern of wheat root growth reported here, with patterns of mature root system architecture and ultimately yield. However, data from our system support the findings that soil-grown wheat roots were shorter in HN conditions compared to LN [44]. Furthermore, this system qualitatively recapitulated results from a field trial on tillering wheat by [45]; growing wheat together shortens their roots. The RSA patterns described here may represent good indicators of susceptibility to blackgrass infestation, and as such could be efficiently scored early and included in breeding programs [46].

### The presence of neighbours affects wheat RSA at early stages

Our data show in detail the effect of neighbouring plants on wheat RSA, in particular we were able to capture changes at the lateral root level that would be masked if only root weight had been measured. Here, similarly to the common bean [17], wheat RSA exhibited greater plasticity than root biomass in response to the presence of a neighbour, especially in HN. Given that thus far no general pattern of root to root interaction has been identified over many different studies [16–25] and that a side by side comparison of 20 species from grasslands found no consistent behavioural response to neighbours [15], it is worthwhile to conduct a species-specific study like ours particularly when focusing on species of agricultural importance.

Length of wheat seminal roots was mainly affected by the presence of another wheat plant while the presence of blackgrass or *Brachypodium* affected lateral roots. In HN, seminal and lateral roots differ in their role in plant performance. Seminals are better able to penetrate the soil because of their higher diameter (S1a and S1d Fig), controlling rooting depth and establishing anchorage. In the field, the presence of a wheat neighbour is likely to affect the rooting depth of wheat plants, which itself will affect tolerance to drought conditions [47]. Wheat laterals, which tend to be thinner (S2b and S2d Fig), are likely to take up water and nutrients at higher rates, as was demonstrated for *Citrus volkameriana* [48]. In addition, the presence of a non-wheat neighbour, by reducing the average length of laterals, will likely lower the total nutrient uptake in wheat. At this point, it is not clear whether the lateral root growth is arrested completely or whether it is slowed down. Here, both roots and shoots could interact thus leading to changes in RSA and it is not clear whether interactions above and/or below ground are causing the observed effects on RSA.

### The wheat response to the presence of a neighbour was different under LN and HN conditions

Nutrient availability has been shown to affect plant interactions [20,21,49]. Our system revealed the importance of nutrition to the wheat response to the presence of a neighbouring weed or crop plant. In HN, mean first order lateral root length was decreased by 30.5% in the blackgrass and 34.3% in the presence of *Brachypodium*, in contrast to LN where there were no suppressive effects on lateral root growth. This is interesting as in LN, lateral roots were longer and more likely to grow closer to a neighbour.

Thus, when a wheat plant is nutrient replete it may be more sensitive, in terms of RSA, to the presence of a neighbour than when it is nutrient deficient. *Albutilon theophrasti* was shown to non-additively integrate information about nutrient availability and the presence of a neighbour [20], suggesting that there is a mechanism by which nutrient level can affect the detection and/or response to a neighbour. In our setting, the interaction of patch nutrient level and neighbour did not significantly affect wheat RSA, *i*.*e*., the level of nutrients available to the neighbour did not overall modulate the neighbour effect on wheat RSA. This suggests that overall the level of nutrients available to the focal plant may be more important than the nutrient level available to the neighbour in determining the neighbour effect.

### The presence of blackgrass affects wheat RSA, mainly lateral root length

Our data show that while in homogeneous HN there was a strong suppressive effect of blackgrass presence on wheat first order lateral root length, this was not the case under a homogeneous LN regime. These data support results from a pot experiment, where blackgrass could suppress more wheat growth under high nitrogen conditions compared to low nitrogen [12], but roots were not specifically analyzed in that study. For other species, nutrient availability was also shown to alter root interactions [20,21,31]. Here, that blackgrass effected inhibition of wheat first order lateral root growth by 30.5% compared to no-neighbour in high nutrients but not in low nutrients lends credence to the idea that weeds are indeed adapted to agronomic, fertilised soils [12]. It also suggests that the nutrient status of the wheat may affect its sensitivity to blackgrass, though further analysis of plant nutritional status would need to be performed to confirm this. Moreover, results here show that blackgrass exerts effects on wheat roots very early on in growth. In order to develop wheat varieties that are better adapted to tolerate the presence of weeds in the field, it is important to know which root traits are valuable to improve productivity in the presence of a neighbour. Under our setting the root trait that is most affected by the presence of a neighbour is the length of lateral roots. Lateral root growth is important for wheat yield [50] and this effect may help explain the impact of blackgrass in the field. Given that Hereward is a variety that is known to be sensitive to blackgrass infestation in the field, perhaps varieties that do not have impaired lateral root growth in response to the presence of blackgrass are likely to overall produce high yield even under heavy weed pressure. Thus, wheat varieties that can maintain good root density (amount of root per unit soil) under low fertilizer input [51] may also prove to be less sensitive to blackgrass infestation.

In comparison with blackgrass, *Brachypodium* was also grown in combination with wheat to test whether it could serve as a tractable model for future molecular studies. *Brachypodium* had similar effect to blackgrass on the wheat seedlings that grew alongside its already established roots, in terms of both FW and RSA in HN levels. Interestingly, *Brachypodium* biomass was lower than that of blackgrass in all experiments. In addition, second order laterals tended to be unaffected by the presence of *Brachypodium* whereas blackgrass presence had a positive effect on their length. Overall, results here show that while *Brachypodium* can exert effects on wheat roots, its effects do not mimic exactly the presence of blackgrass suggesting that some of the effects observed here may be species-specific.

## Conclusions

To prevent yield loss due to weed infestation, new sustainable methods must be explored as we cannot solely rely on the development of new chemicals or change in agronomic practices. Understanding the effect of weeds on crops may lead to the development of crops that are more tolerant of the presence of neighbours from weed species. Our study suggests that in terms of root length wheat is more sensitive to the presence of a neighbour when nutrient availability is not limited. Thus the conditions that farmers try to achieve with the addition of fertilisers may enhance the negative effect of blackgrass. This makes exploring the mechanism underlying the neighbour effect highly relevant to the development of new crop varieties. While genotypic variation in wheat for RSA have been described [42,52,53] genetic variability in root response to the presence of a weed neighbour, particularly blackgrass, would be worth further exploration.

## Materials and methods

### Plant material

Seeds of the winter wheat variety ‘Hereward’ were obtained from Hutchinson Ltd. (UK). Blackgrass seeds were collected from the Broadbalk experiment (gift from Dr. S. Moss, Rothamsted Research, UK). *Brachypodium dystachyon* seeds were from the BD21 ecotype (gift from Dr. P. Vain, John Innes Centre, UK).

### Growth conditions

Seeds from wheat, blackgrass and *Brachypodium* were surface sterilised by incubation for 5 min with 70% (v/v) ethanol, rinsing with sterile distilled water, followed by 15 min incubation with 10% (v/v) sodium hypochlorite and three rinses with sterile distilled water. Wheat seeds were germinated on plates filled with 0.8% (w/v) phytagel (Sigma-Aldrich, USA) and half-strength Murashige-Skoog (MS) medium (Duchefa, Netherlands), while blackgrass and *Brachypodium* seeds were germinated in magenta vessels (width: 77mm, length: 77mm, height: 97mm) filled with 400 mL of MS medium and covered with magenta vessels of the same dimension. Magenta vessels, bases and lids, were secured by couplers. Magenta vessels were randomly placed in a growth chamber where conditions were as follow: 25°C, 16h light: 8h dark, and light intensity of 78 μmol m^-2^ s^-1^.

### Competition in high nutrient conditions

Wheat plants were grown alone (w) or in the presence of another wheat plant (w-w), a blackgrass (bg) or a *Brachypodium* individual (bd). Blackgrass or *Brachypodium* plants were sown 11 days ahead of the wheat plants in a magenta vessel containing full strength MS medium (pH 5.7 with KOH) with 0.12% (w/v) phytagel (High nutrient medium, HN; Fig 1a). This was so the fresh weight (FW) of the neighbouring grass was similar to that of the wheat neighbour. Wheat seeds (both focal and neighbour plants) were germinated on plates (half-strength MS medium, 0.8% (w/v) phytagel) for three days, until the first leaf was visible, then transferred to magenta vessels under sterile conditions, taking care not to damage the roots. In all competition treatments, the focal wheat plant and neighbours (wheat, bg or bd) were spaced approximately one cm apart (Fig 1a). All plants were harvested 11–13 days later, FW was measured for both shoot and root. The root system could easily be extracted from the phytagel for analysis of its architecture.

### Competition in patchy nutrient conditions

As before, the focal wheat plant was grown alone (w) or in the presence of another wheat plant (w-w), a blackgrass (bg) or a *Brachypodium* individual (bd) (Fig 1e). In this set of experiments, the focal wheat plant was grown in low nutrient (LN) conditions (1/100 MS supplemented with 3 mM CaCl_2_.2H_2_O and 0.75 mM MgCl_2_.6H_2_O to help the gelling agent to set (0.12% (w/v) phytagel) while the competitor (w, bg or bd) was placed in a patch of either low (LN, 1/100 MS) or high nutrient (HN, MS as described previously; Fig 1e). Nutrient patches were created in the medium by inserting a sterile tube (30 mm diameter) in the magenta, pouring medium around it, removing the tube when the medium was set and filling in with LN or HN medium (added as sterile liquid and allowed to set). Blackgrass and *Brachypodium* neighbours were grown for 11 days in the patch before adding germinating wheat seeds (as before these were germinated on half-strength MS medium, 0.8% (w/v) phytagel). All plants were collected 11 days after the addition of wheat.

### Root system architecture (RSA) analysis

Plant roots were collected from the gelling agent, rinsed in distilled water and placed in a clear plastic plate with distilled water before scanning on a flat-bed scanner at a resolution of 300 dpi (Fig 1c). Detailed analysis of wheat RSA was conducted using SmartRoot [36] a plugin for ImageJ [54], and RSA traits are described in Table 1. SmartRoot represents an individual root as a vector or set of segments connected through nodes (Fig 1d, nodes are represented as circles along the roots). The root diameter is estimated at each node and averaged along the root. The number of nodes (and segments) varies along the root, for example to accurately measure the length it is higher in areas where the root is curved. The nodes are also used as anchors where vectors representing first order laterals can be virtually attached to the vector representing the seminal root (from which the laterals emerge). Since the position along the root of each node is recorded, the positions of lateral root on the seminal root are also available. Here the distance between the base of the root and the first lateral root emerging (in cm) was divided by the root length (in cm) to calculate the relative position of the first lateral and allow comparison amongst roots of different length. A similar calculation was conducted to obtain the relative position of the last lateral root. From the individual root length data provided by SmartRoot, cumulative length of seminals or laterals was calculated by adding the length of each root from a specific category (either seminal or lateral) from the same image. Frequency distribution or density plots (function qplot with argument geom = "density", in R (R Core Team, 2012)) were constructed to display the distribution of lateral root length (Figs 3b, 3d, 6a and 6b).

### Statistical analyses

Statistical analyses were conducted in R. Normal distribution of data and equality of variance were checked using Shapiro and Levene’s (*lawstats* package) tests respectively. When these assumptions were upheld, analyses were conducted on raw or transformed data (log or square root), otherwise analyses were conducted on rank values. In the first set of experiments (effect of neighbour in HN), Student’s *t*-test was used to compare the effect of a different neighbour on wheat FW. While in the second set of experiments (effect of neighbour in LN with LN/HN patch), two-way ANOVAs were conducted using Neighbour and Patch as fixed effects, including their interactions. Tukey HSD test was used to test the significance between levels of the fixed effect tested.

Where multiple roots were individually measured per individual plant (*e*.*g*. mean length), linear mixed effect analysis was conducted using the *nlme* [55] package to assess the treatment effect on root traits (Table 1). In the first set of experiment (effect of neighbour under HN conditions), the model was constructed to include neighbour (w, w-w, w-bg, w-bd) as a fixed effect. In the second set of experiments (effect of neighbour under LN with LN/HN patch), the model was constructed to include neighbour (w, w-w, w-bg, w-bd) and patch (LN, HN) as a fixed effect including their interactions. Since multiple roots were measured on each plant, intercept for an individual plant was included as a random effect. Standardized residuals were plotted against fitted values and indicated no obvious deviation from homoscedasticity or normality. ANOVA of the linear mixed-effect model run using the maximum likelihood ('ML') instead of 'REML' was used to obtain an overall *p*-value, while *p*-values relating to comparisons amongst treatments were obtained from the lme model summary. The Kolmogorov-Smirnov test was applied to compare the distribution of laterals and the *p* value was reported in the text. Nutrients effects and interaction between nutrients and the presence of a neighbour were tested by either two-way ANOVA or lme.

## Supporting information

S1 Fig Average root diameter of wheat seminals and laterals grown under different nutrient and competitive conditions. (a) Seminal diameter of individual roots (black filled circles) and overall mean (red diamonds) of plants grown in HN. (b) First order laterals diameter of plants grown in HN. (c) Second order laterals diameter of plants grown in HN. (d) Seminal and (e) first order lateral diameter of wheat plants grow in LN in the presence of a neighbour grown in either a HN or LN patch. (w, wheat alone; w-w, wheat against wheat; w-bd, wheat against *Brachypodium*; w-bg, wheat against blackgrass). For experiments in HN (a, b, c);^**.**^ *p* < 0.1, * *p* < 0.05. For experiments in LN (d, e), significance levels of main factors (N, neighbour, P, patch) and their interactions (N x P) are shown;^**.**^ *p* < 0.1, * *p* < 0.05, *n*.*s*., not significant.
(TIF)

S2 Fig Relative position of the laterals closest (first) or furthest (last) to the base of the root. Position calculated by SmartRoot was divided by the length of the root to enable comparisons across roots of different length. Each individual black filled circle corresponds to a root, while averages for each treatment are represented by red diamonds. (a) Relative position of first lateral on seminal root grown in HN. (b) Relative position of last lateral on seminal root grown in HN. (c) Relative position of first lateral on seminal root grown in LN. (d) Relative position of last lateral on seminal root grown in LN. (w, wheat alone; w-w, wheat against wheat; w-bd, wheat against *Brachypodium*; w-bg, wheat against blackgrass). For experiments in HN (a, b),^**.**^ *p* < 0.1, and * *p* < 0.05. For experiments in LN (c, d), significance levels of main factors (N, neighbour, P, patch) and their interactions (N x P) are shown,^**.**^ *p* < 0.1, * *p* < 0.05, *n*.*s*., not significant.
(TIF)

S1 Table Root system architecture of plants grown in high nutrients. (CSV)

S2 Table Total fresh weight of focal wheat plants grown in high nutrients. (CSV)

S3 Table Total fresh weight of neighbour plants grown in high nutrients. (CSV)

S4 Table Root system architecture of plants grown in low nutrients with low and high nutrient patches. (CSV)

S5 Table Total fresh weight of focal wheat plants grown in low nutrients with low and high nutrient patches. (CSV)

S6 Table Total fresh weight of neighbour plants grown in low nutrients with low and high nutrient patches. (CSV)
