# Supplementary figures and images for: Wheat root length and not branching is altered in the presence of neighbours, including blackgrass
Source: PLoS One. 2017 May 24;12(5):e0178176. doi: 10.1371/journal.pone.0178176 (PMC5443546; doi:10.1371/journal.pone.0178176)

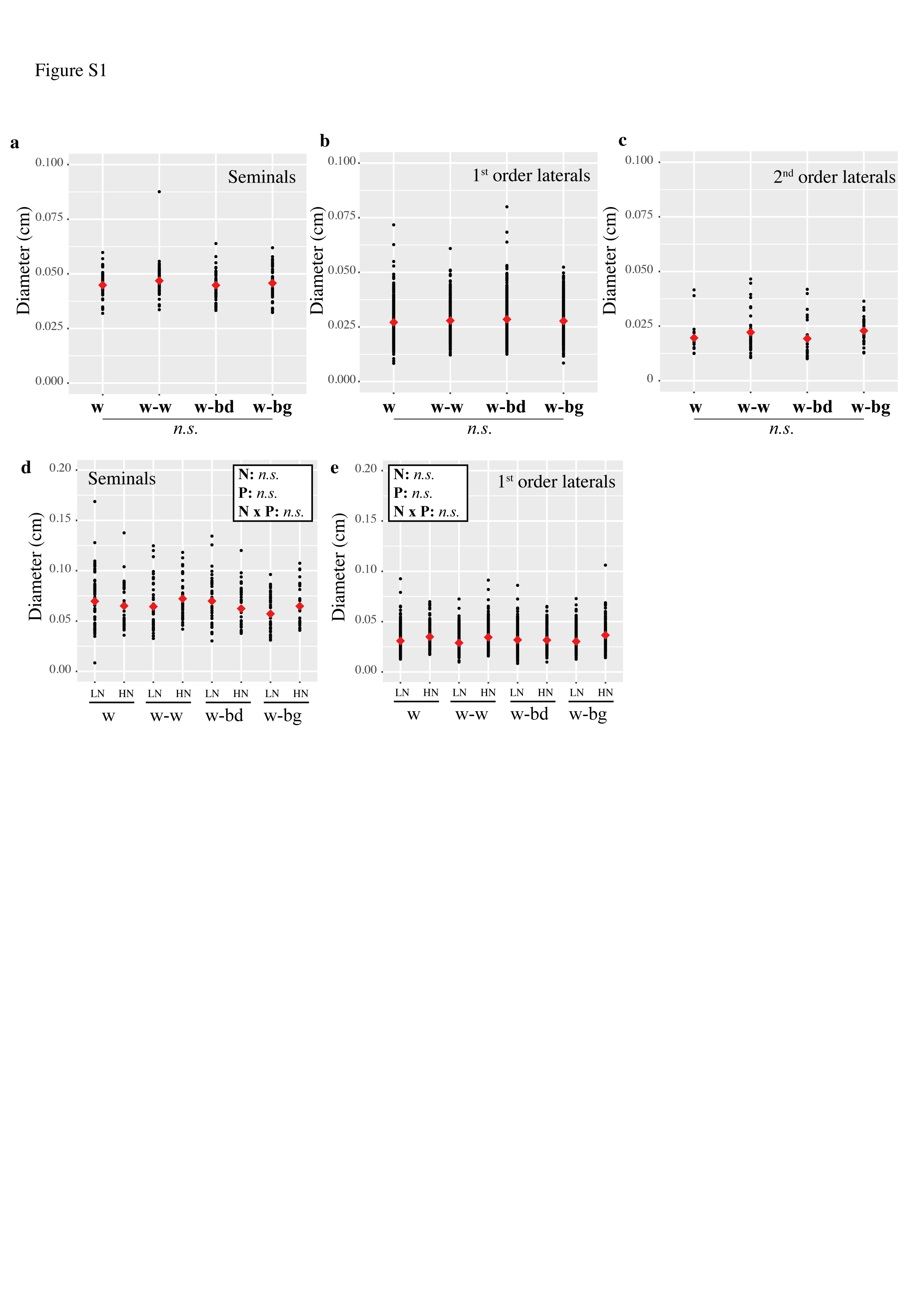

Supplement: S1 Fig — (a) Seminal diameter of individual roots (black filled circles) and overall mean (red diamonds) of plants grown in HN. (b) First order laterals diameter of plants grown in HN. (c) Second order laterals diameter of plants grown in HN. (d) Seminal and (e) first order lateral diameter of wheat plants grow in LN in the presence of a neighbour grown in either a HN or LN patch. (w, wheat alone; w-w, wheat against wheat; w-bd, wheat against Brachypodium; w-bg, wheat against blackgrass). For experiments in HN (a, b, c);. p < 0.1, * p < 0.05. For experiments in LN (d, e), significance levels of main factors (N, neighbour, P, patch) and their interactions (N x P) are shown;. p < 0.1, * p < 0.05, n.s., not significant. (TIF) [file pone.0178176.s001.tif]

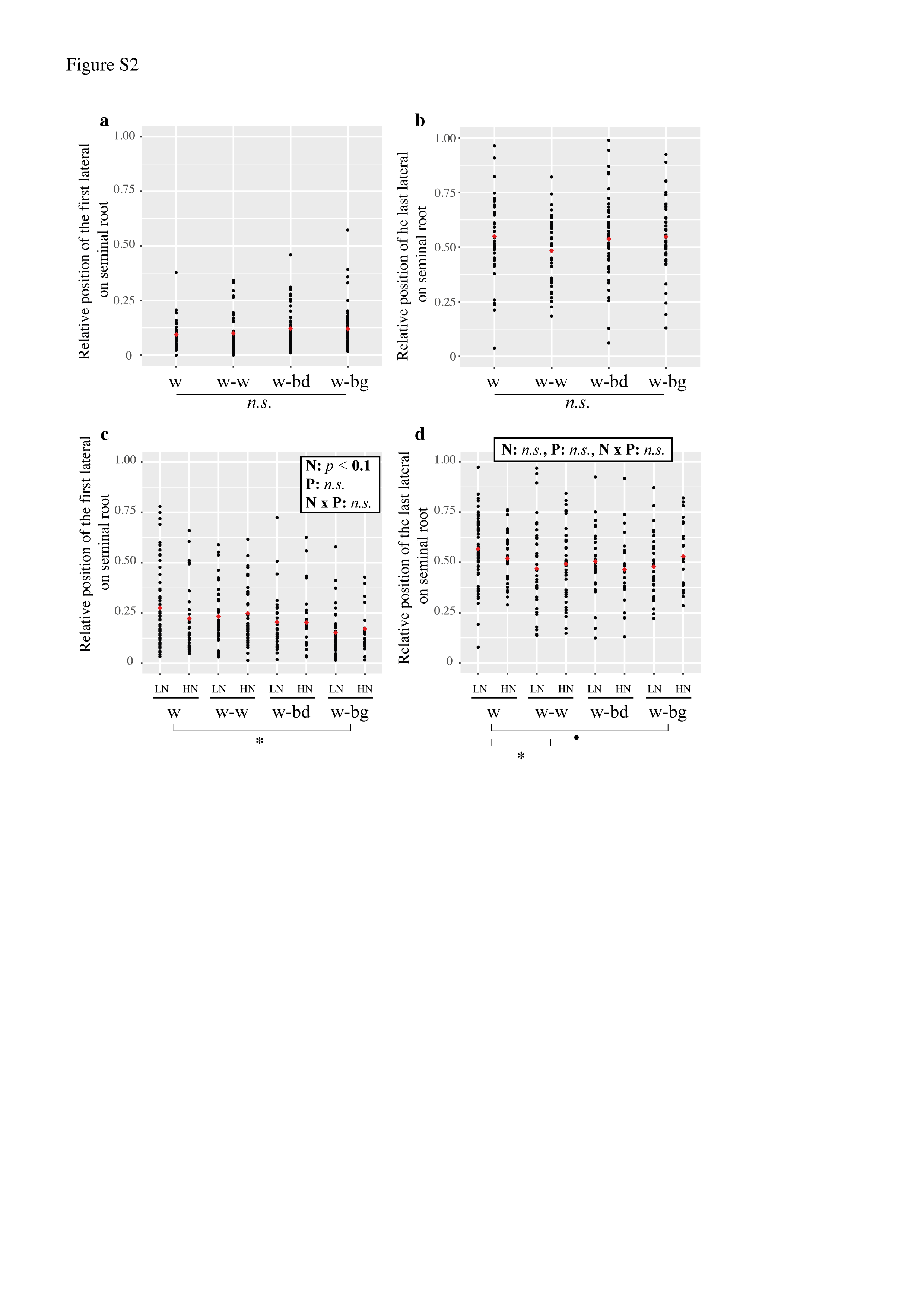

Supplement: S2 Fig — Position calculated by SmartRoot was divided by the length of the root to enable comparisons across roots of different length. Each individual black filled circle corresponds to a root, while averages for each treatment are represented by red diamonds. (a) Relative position of first lateral on seminal root grown in HN. (b) Relative position of last lateral on seminal root grown in HN. (c) Relative position of first lateral on seminal root grown in LN. (d) Relative position of last lateral on seminal root grown in LN. (w, wheat alone; w-w, wheat against wheat; w-bd, wheat against Brachypodium; w-bg, wheat against blackgrass). For experiments in HN (a, b),. p < 0.1, and * p < 0.05. For experiments in LN (c, d), significance levels of main factors (N, neighbour, P, patch) and their interactions (N x P) are shown,. p < 0.1, * p < 0.05, n.s., not significant. (TIF) [file pone.0178176.s002.tif]
